# Association Between Internet Searches Related to Suicide/Self-harm and Adolescent Suicide Death in South Korea in 2016-2020: Secondary Data Analysis

**DOI:** 10.2196/46254

**Published:** 2023-04-20

**Authors:** Won-Seok Choi, Junhee Han, Hyun Ju Hong

**Affiliations:** 1 Department of Psychiatry, Yeouido St Mary’s Hospital, College of Medicine, The Catholic University of Korea Seoul Republic of Korea; 2 Department of Statistics, Hallym University Chunchon Republic of Korea; 3 Department of Psychiatry, Hallym University Sacred Heart Hospital, College of Medicine, Hallym Univerisity Anyang Republic of Korea

**Keywords:** adolescent, suicide, self-mutilation, internet, search engine, Korea, suicide death, surveillance, monitoring, internet search

## Abstract

**Background:**

Previous studies have investigated the association between suicide and internet search volumes of terms related to suicide or self-harm. However, the results varied by people’s age, period, and country, and no study has exclusively investigated suicide or self-harm rates among adolescents.

**Objective:**

This study aims to determine the association between the internet search volumes of terms related to suicide/self-harm and the number of suicides among South Korean adolescents. We investigated gender differences in this association and the time lag between the internet search volumes of the terms and the connected suicide deaths.

**Methods:**

We selected 26 search terms related to suicide and self-harm among South Korean adolescents, and the search volumes of these terms for adolescents aged 13-18 years were obtained from the leading internet search engine in South Korea (Naver Datalab). A data set was constructed by combining data from Naver Datalab and the number of suicide deaths of adolescents on a daily basis from January 1, 2016, to December 31, 2020. Spearman rank correlation and multivariate Poisson regression analyses were performed to identify the association between the search volumes of the terms and the suicide deaths during that period. The time lag between suicide death and the increasing trend in the search volumes of the related terms was estimated from the cross-correlation coefficients.

**Results:**

Significant correlations were observed within the search volumes of the 26 terms related to suicide/self-harm. The internet search volumes of several terms were associated with the number of suicide deaths among South Korean adolescents, and this association differed by gender. The search volume for “dropout” showed a statistically significant correlation with the number of suicides in all adolescent population groups. The correlation between the internet search volume for “dropout” and the connected suicide deaths was the strongest for a time lag of 0 days. In females, self-harm and academic score showed significant associations with suicide deaths, but academic score showed a negative correlation, and the time lags with the strongest correlations were 0 and –11 days, respectively. In the total population, self-harm and suicide method were associated with the number of suicides, and the time lags with the strongest correlations were +7 and 0 days, respectively.

**Conclusions:**

This study identifies a correlation between suicides and internet search volumes related to suicide/self-harm among South Korean adolescents, but the relatively weak correlation (incidence rate ratio 0.990-1.068) should be interpreted with caution.

## Introduction

Suicide is a leading contributor to disease burden and deaths globally, with almost 1 million suicide deaths across the world annually [1]. In particular, suicide is the second leading cause of death globally among young people aged 10-24 years [2]. In South Korea, suicide has been the leading cause of death among children and youths aged 9-24 years for decades, thereby representing a notable social burden on the society [3]. Web-based spaces are now part of our daily lives, and they can be utilized as suicide surveillance systems, especially for adolescents. Most European adolescents (92%) have at least one social networking service account, and 40% of them spend more than 2 hours a day online [4,5]. Individuals who plan to die by suicide often use the internet to share their thoughts of suicide or to find ways to die by suicide [6,7].

Previous studies have investigated the association between suicide rates and the internet search volumes of terms related to suicide or self-harm [8-14]. However, the results varied by age, period, and country, and no study has exclusively investigated suicide/self-harm rates among adolescents. A study conducted in Taiwan [10] using Google Trends as a search engine revealed that the internet search volumes of suicide-related terms were associated with actual suicide deaths. An Irish study using Google Trends [14] found an association between the national suicide rate and the internet search volume of suicide-related terms, including anxiety. A study that matched internet search activity to national suicide rates according to Google Trends found a negative correlation with suicide rates in the general US population but a positive correlation with suicide rates in youth (age 15-24 years) [8]. Google Trends was the most frequently used search engine in the abovementioned studies, and it quantifies search trends by using the relative search volume (RSV) of each term. The RSV ranges from 0 to 100, corresponding to the number of searches for a given term as a proportion of the largest number of searches over a given period; for example, an RSV of 30 equates to 30% of the most popular search activity at any time. However, Google Trends can only provide RSVs averaged over at least 1 week, and it does not provide information on gender difference. Moreover, the shortest time period reported using Google Trends in previous studies on the relationship between the internet search volumes for specific terms and the actual suicide death was 1 month [9,10,13-15]. However, adolescent suicides are more impulsive than those of adults; for example, the number of suicides was found to increase rapidly at 1-10 days after the suicide of a celebrity [16,17]. Furthermore, in our preliminary study of internet search volumes and adolescent suicide death, which has not yet been published, the time interval with the strongest relationship between internet search volume and suicide was less than 2 weeks. These observations make it likely that the time interval between the actual internet search activity for terms related to suicide/self-harm and an actual suicide death is less than 2 weeks.

In South Korea, Naver [18] is the largest and the most popular search engine, with Google being less popular [19]. Naver Datalab [20] is a free service that provides the search volumes and trends of specific terms in the same way as Google Trends. However, unlike in Google Trends, in Naver Datalab, it is possible to extract the search volume of a given term for each day according to the RSV. Moreover, by collecting demographic data, including the age and gender of the logged-in users, the search volume can be subdivided by gender and age using this service. The RSV data extracted from Naver Datalab have been widely used in the research of search volumes in South Korea, such as for predicting the outbreak of infectious diseases [21,22] and for calculating the mediating effect of gender in depression and suicide after COVID-19 [23]. The 2 aims of our study based on the analysis of Naver Datalab and South Korean national data of all 13-18-year-old students who died by suicide in 2016-2020 on a daily basis were (1) to identify any association between the internet search volumes of terms related to suicide/self-harm and the number of suicide deaths according to gender and (2) to estimate the specific time lag presenting the strongest correlation for this association.

## Methods

### Database of Student Suicides

In South Korea, when a student dies by suicide, teachers are required to report this to the Ministry of Education by using a form called the Student Suicide Report. Thus, there is a data set of all suicides of South Korean students attending elementary, middle, and high schools. Detailed information on the processing and contents of the Student Suicide Report has been published previously [24]. We extracted data on the gender and death dates of adolescents aged 13-18 years who died by suicide from January 1, 2016, to December 31, 2020, from this database. Considering that the dropout rate of South Korean high school students was about 1.5% [25], this proportion can represent the most proportion of adolescent suicides in South Korea. In case where the date on which the body was found was recorded but the date of death was unknown, the date on which the body was found was regarded as the date of death. Since about 90% of the suicide methods among South Korean adolescents who died by suicide were fatal (eg, jumping from a height, hanging) [24], it was assumed that there was a short time interval between the date of death and the date of discovery. We excluded cases with no information or where the date of death based on the above criteria was unknown, and we finally included 620 cases of suicide among 625 students aged 13-18 years during the study analysis period.

### Search Terms Related to Suicide and Self-harm

The temporal association between the number of suicides and trends in the internet search volumes of terms related to suicide/self-harm was investigated by selecting appropriate search terms related to suicide and self-harm among South Korean adolescents. We selected 26 search terms based on previous studies of search terms related to suicide [8-13] and South Korean studies on the risk factors for suicide [26-29], methods of suicide [24,30], and self-harm [31] among adolescents. In this process, search terms with similar meanings were classified into search terms considering the characteristics of the South Korean population. For example, the search term “suicidal method” could be searched as “how to suicide” and “way to suicide,” and the search terms for “suicide method” included “how to suicide,” “way to suicide,” and “suicide method.” Like Google, Naver has a system that warns users when searching for words related to suicide or blocks dangerous sites [32]. However, Naver does not automatically search and collect RSVs for synonyms like Google; therefore, we acquired the search volume of each search term together. Then, referring to previous studies [10,14], we classified the search terms into 5 groups: (1) suicide-related terms, (2) self-harm–related terms, (3) suicide risk factor terms, (4) suicide prevention terms, and (5) depression-related terms. All the selected search terms were searched in Korean. Table 1 lists the groups of search terms translated into standard English and the related synonyms (see Multimedia Appendix 1 for the standard Korean versions of the search terms).

**Table 1:** Search terms related to suicide and self-harm.

Classification of search terms, search terms		Synonyms
**Suicide-related terms**		
	Suicide	Suicide
	Suicide method	Suicide method, how to suicide, how to die by suicide
	Dying method	Dying method, how to die
	Suicidal idea	Suicidal idea, suicidal thinking
	Fall down	Fall down, suicide by jumping from a height
	Hanging	Hanging, hanging suicide, neck hanging
	Will	Will, how to write a will
**Self-harm–related terms**		
	Self-harm	Self-harm
	Self-harm method	Self-harm method, how to self-harm
	Wrist cutting	Wrist cutting, how to cut my wrist, wrist-cutting method
	Self-harm wound	Self-harm wound, self-harm mark, treatment for self-harm wound
	Drug overdose	Drug overdose, drug lethal dose
	Acetaminophen	Acetaminophen overdose, acetaminophen lethal dose
**Suicide risk factor terms**		
	Academic score	Academic score, academic concern
	Bullying	Bullying, covert bullying, outcast
	School violence	School violence, school vio^a^
	Family troubles	Family troubles
	Domestic violence	Domestic violence
	Dropout	Dropout, how to drop out, dropout method
	Career	Career, career concern
**Suicide prevention terms**		
	Suicide prevention	Suicide prevention
	Call for life	Call for life, call for life of South Korea
	Suicide prevention center	Suicide prevention center, 1393^b^
	Psychiatry	Psychiatry, neuropsychiatry, psychiatry department, mental hospital
	Mental health center	Mental health center
**Depression-related term**		
	Depression	Depression, depressed, depressive disorder, depressive symptoms

^a^An abbreviated search term for school violence.

^b^National Suicide Prevention helpline in South Korea.

### Source of Internet Search Volume Data

The data on suicide and the volume of internet search terms for self-harm were obtained from Naver Datalab [20] on May 3, 2022. Daily RSVs of 26 suicide and self-harm–related terms searched by adolescents aged 13-18 years were obtained from January 1, 2016, to December 31, 2020. When there were multiple synonyms for a single search term, all search volumes of these synonyms were included in the RSV for that search term. In addition, the RSVs were obtained separately according to gender. The search volumes in this study include all searches performed using personal computers and mobile devices.

### Statistical Analyses

A data set was constructed by combining data from Naver Datalab and the number of suicide deaths of adolescents on a daily basis from January 1, 2016, to December 31, 2020, for statistical analyses. First, considering the nonnormality of the variables, we performed Spearman rank correlation analysis to assess the associations between each RSV for suicide and self-harm–related search terms in the male, female, and total populations. Second, considering that the sum of the total suicides per day ranges from 0 to 4, we employed a multivariate Poisson regression model to estimate the association by using search volumes of search terms as independent variables and daily suicide deaths as the dependent variable.

To estimate the time lag of the increasing trend between suicide death and search volumes, our analysis proceeded in 2 steps. First, to identify the most relevant time lag, we performed Poisson regression analyses of the association between the number of suicides and search volumes repeatedly with different time intervals of 1 day, 5 days, 10 days, and 15 days. Variables included in each Poisson regression analysis were synthesized as the sum of the number of suicides and the search volume for each time interval. Thereafter, the regression coefficient (r) was calculated for the correlation between the number of suicides and the search volume for the search term during each of the 4 time intervals, and the time interval for which the regression coefficient was statistically significant was identified. If a statistically significant regression coefficient was obtained for multiple time intervals for a single search term, we assumed that there would be a time lag with the strongest correlation between the number of suicides and the search volumes of terms related to suicide/self-harm between the time interval at which the highest and the second highest regression coefficients were obtained. Second, to identify a more accurate time lag, we assumed that the time interval with the strongest correlation would exist between 2 time intervals that were statistically significant. Thus, a cross-correlation analysis of the correlation between the daily number of suicides and the search volume was performed for the search terms identified in the first step. This approach has been used in previous studies [9,10] to investigate the association between suicide and internet search and to identify the time lag of the increasing trend. The time lag showing the highest correlation coefficient for the 2 time intervals was identified. If a positive time lag was identified, it means that changes in internet search precede changes in suicide deaths. Conversely, if a negative time lag was identified, it meant the opposite. R software (version 4.2.0; R Core Team and the R Foundation for Statistical Computing) was used for the statistical analyses. Significance in all statistical analyses was defined as *P*<.05.

### Ethics Approval

Ethics approval for this study was obtained from the Hallym Sacred Heart Hospital institutional review board (approval HALLYM 2020-08-008). Patient consent was waived because this study was the secondary analysis of existing data. The data were collected as part of National Student Suicide Prevention Policy and anonymized by not including personal identification data.

## Results

### Distribution of the Number of Suicides Per Day

The total number of suicides from January 1, 2016, to December 31, 2020, among the adolescents included in this study was 620, comprising 323 male deaths and 297 female deaths. During our research period (a total of 1827 days), the days without any adolescent suicide deaths were 1540 days for males, 1557 days for females, and 1324 days for the total population. The days with 1 adolescent suicide death were 252 days for males, 246 days for females, and 400 days for the total population. The days with 2 adolescent suicide deaths were 34 days for males, 21 days for females, and 92 days for the total population. The days with 3 adolescent suicide deaths were 1 day for males, 3 days for females, and 8 days for the overall population. The days with 4 adolescent suicide deaths were 3 days only for the total population.

### Analysis of Correlation Between RSVs of Search Terms

**Figure 1:**
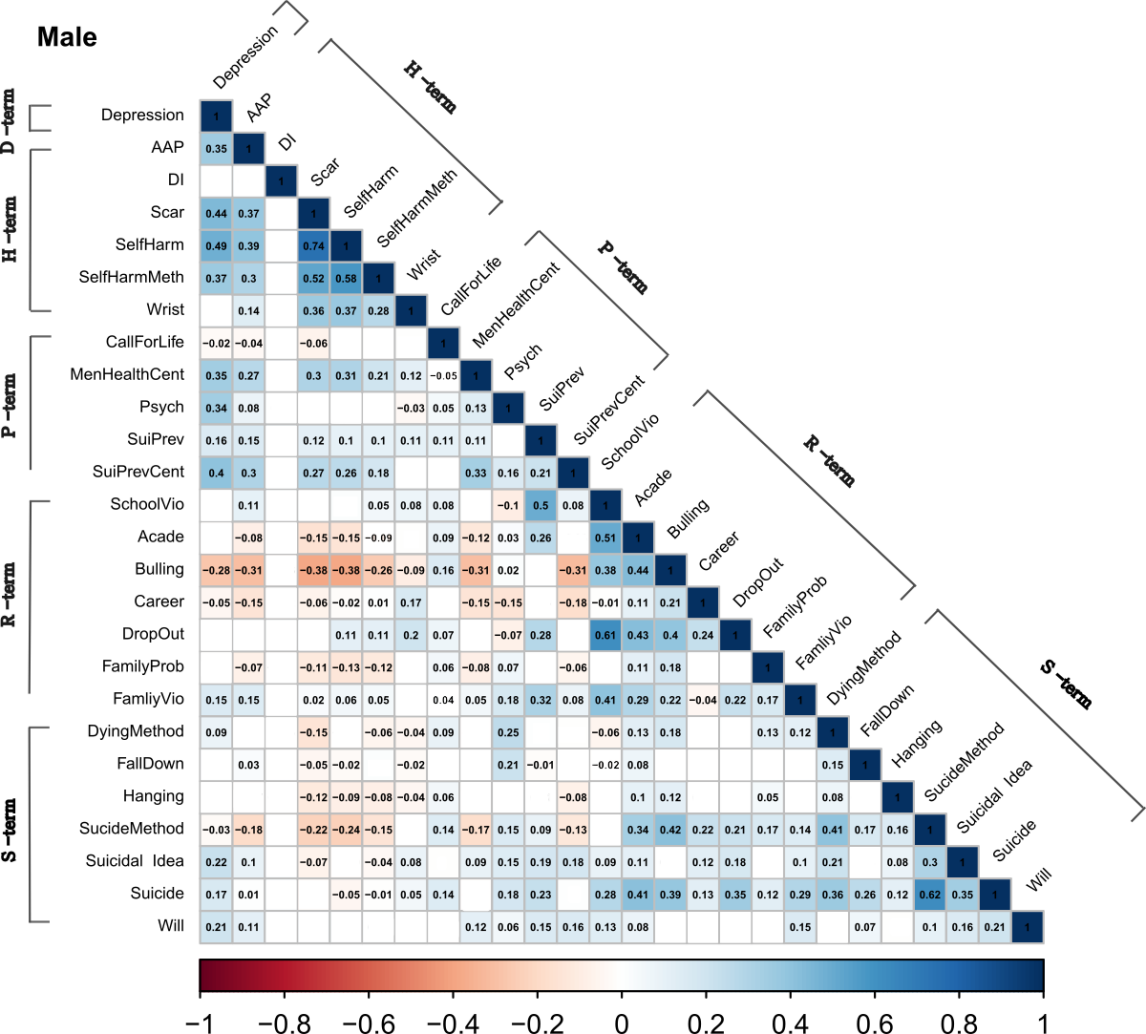
Correlations between the relative search volumes of search terms (male). The blue shade indicates a positive correlation, while the red shade indicates a negative correlation. AAP: acetaminophen; Acade: academic score; Bulling: bullying; DI: drug overdose; D-term: depression-related terms; FamilyProb: family troubles; FamilyVio: domestic violence; H-term: self-harm–related terms; MenHealthCent: mental health center; Psych: psychiatry; P-term: suicide prevention terms; R-term: suicide risk factor terms; SchoolVio: school violence; SelfHarmMeth: self-harm method; S-term: suicide-related terms; SuiPrev: suicide prevention; SuiPrevCent: suicide prevention center; Wrist: wrist cutting.

**Figure 2:**
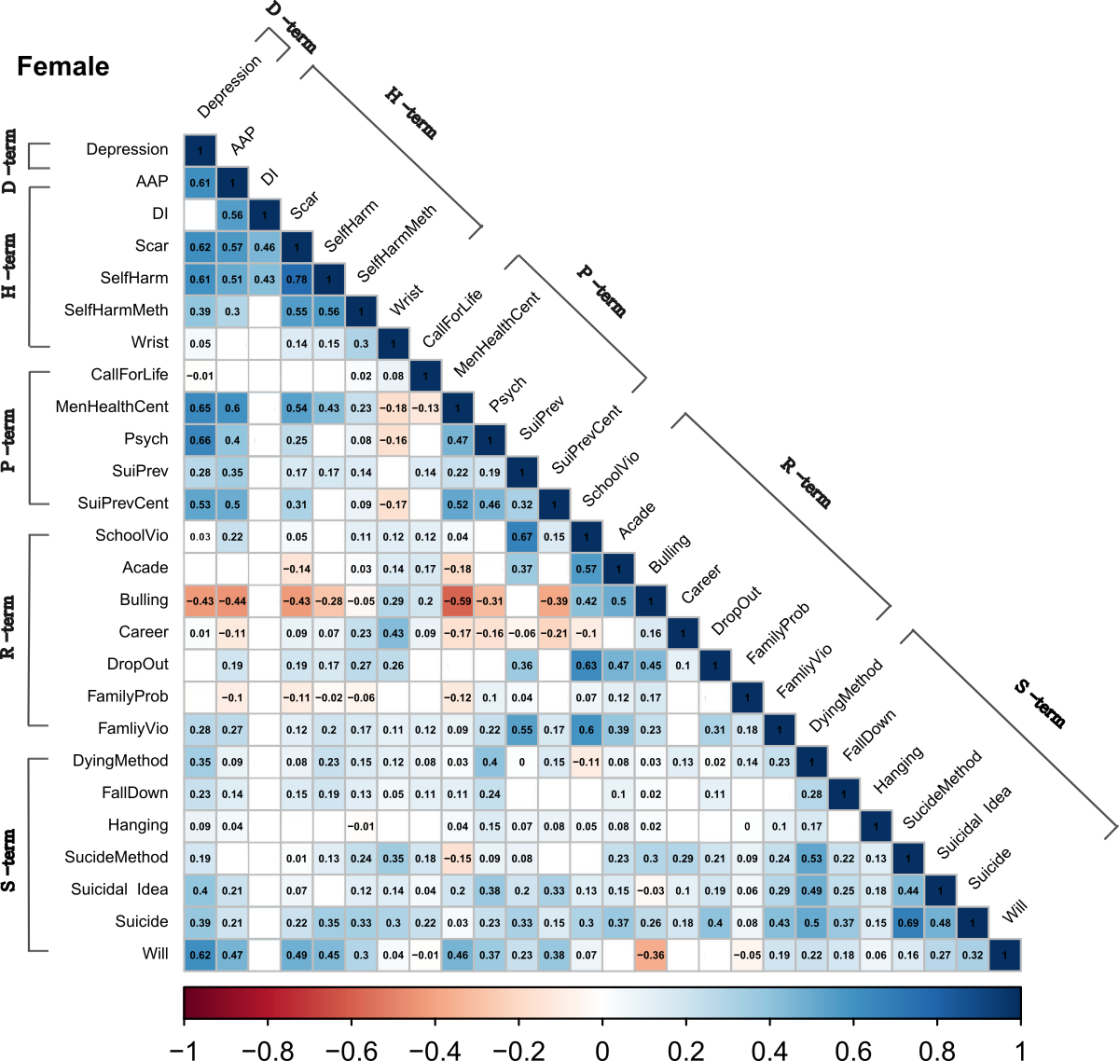
Correlations between the relative search volumes of search terms (female). The blue shade indicates a positive correlation, while the red shade indicates a negative correlation. AAP: acetaminophen; Acade: academic score; Bulling: bullying; DI: drug overdose; D-term: depression-related terms; FamilyProb: family troubles; FamilyVio: domestic violence; H-term: self-harm–related terms; MenHealthCent: mental health center; Psych: psychiatry; P-term: suicide prevention terms; R-term: suicide risk factor terms; SchoolVio: school violence; SelfHarmMeth: self-harm method; S-term: suicide-related terms; SuiPrev: suicide prevention; SuiPrevCent: suicide prevention center; Wrist: wrist cutting.

**Figure 3:**
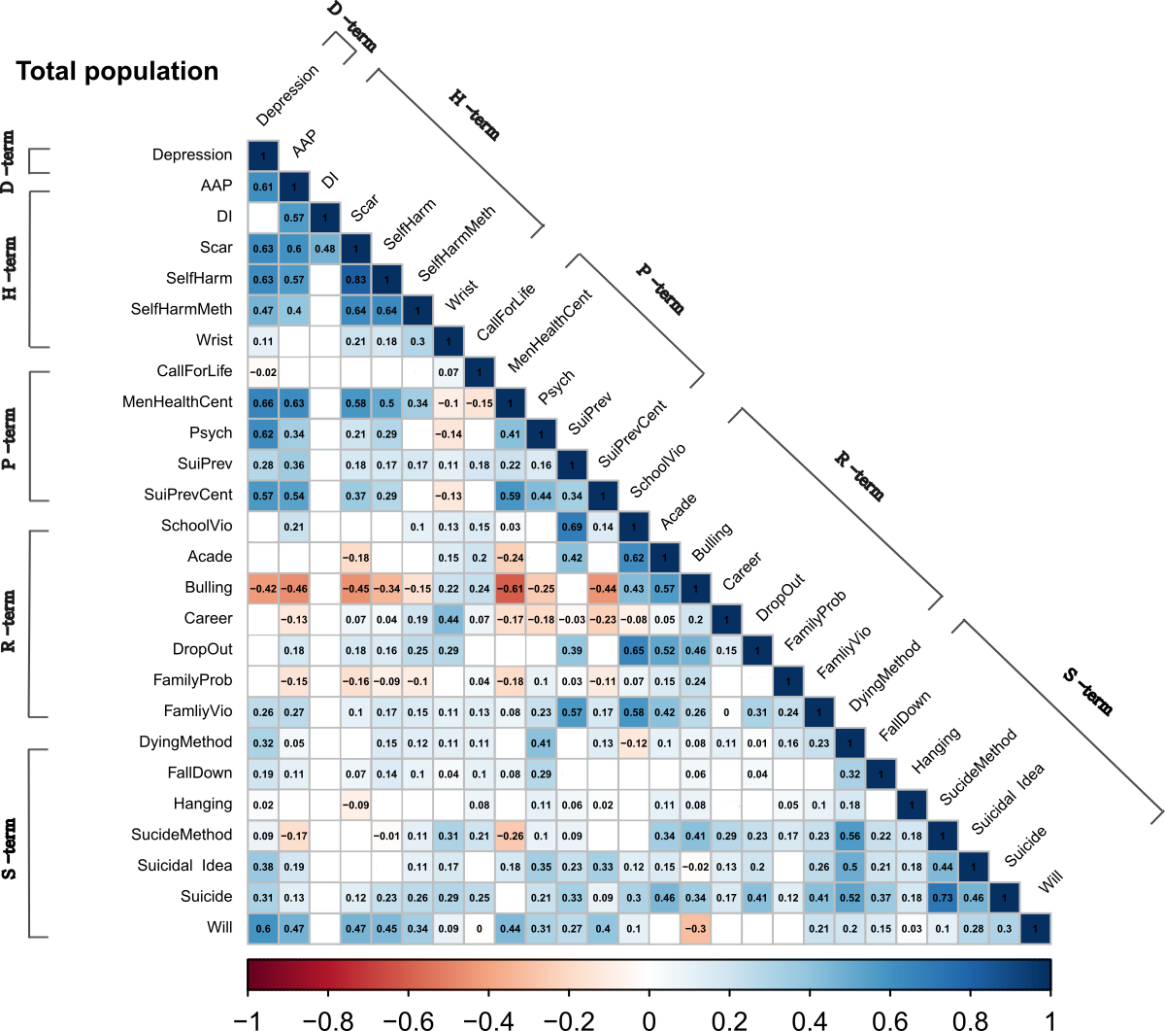
Correlations between the relative search volumes of search terms (total population). The blue shade indicates a positive correlation, while the red shade indicates a negative correlation. AAP: acetaminophen; Acade: academic score; Bulling: bullying; DI: drug overdose; D-term: depression-related terms; FamilyProb: family troubles; FamilyVio: domestic violence; H-term: self-harm–related terms; MenHealthCent: mental health center; Psych: psychiatry; P-term: suicide prevention terms; R-term: suicide risk factor terms; SchoolVio: school violence; SelfHarmMeth: self-harm method; S-term: suicide-related terms; SuiPrev: suicide prevention; SuiPrevCent: suicide prevention center; Wrist: wrist cutting.

In male adolescents, there were statistically significant positive within-group correlations in self-harm–related terms, suicide risk factor terms, and suicide-related terms (Figure 1). The correlation between the overall search terms was stronger in female adolescents than in males, and there were positive within-group correlations for self-harm–related terms, suicide risk factor terms, and suicide-related terms (Figure 2). However, unlike in males, there were positive intergroup correlations between depression-related terms and the other 4 groups of search terms among females. Further, positive intergroup correlations between self-harm–related terms and suicide-related terms or between suicide risk factor terms and suicide-related terms were examined in female adolescents. In the total population, the pattern of the correlations was the same as that in females (Figure 3).

### Poisson Regression Analyses of the Associations Between Suicide Deaths and Search Terms

Table 2 lists the search terms and their incidence rate ratio (IRR) values calculated using Poisson multivariate regression for each time interval. The search terms that showed statistically significant results at multiple time intervals were dropout (IRR 1.021, 95% CI 1.010-1.033; *P*<.001 for a 1-day interval; IRR 1.004, 95% CI 1.000-1.007; *P*=.003 for a 5-day interval) and mental health center (IRR 1.003, 95% CI 1.000-1.006; *P*=.04 for a 10-day interval; IRR 1.003, 95% CI 1.000-1.005; *P*=.04 for a 15-day interval) in males. The search terms self-harm (IRR 1.038, 95% CI 1.009-1.068; *P*=.009 for a 1-day interval; IRR 1.013, 95% CI 1.003-1.022; *P*=.008 for a 5-day interval; IRR 1.008, 95% CI 1.002-1.014; *P*=.01 for a 10-day interval; IRR 1.006, 95% CI 1.000-1.011; *P*=.04 for a 15-day interval), academic score (IRR 0.994, 95% CI 0.990-0.998; *P*=.003 for a 5-day interval; IRR 0.997, 95% CI 0.995-1.000; *P*=.03 for a 10-day interval; IRR 0.997, 95% CI 0.995-0.999; *P*=.005 for a 15-day interval), and dropout (IRR 1.015, 95% CI 1.004-1.025; *P*=.004 for a 1-day interval; IRR 1.001, 95% CI 1.000-1.002; *P*=.049 for a 15-day interval) were identified as being significant in females. In the total population, dropout (IRR 1.017, 95% CI 1.008-1.025; *P*<.001 for a 1-day interval; IRR 1.004, 95% CI 1.001-1.006; *P*=.001 for a 5-day interval; IRR 1.002, 95% CI 1.000-1.003; *P*=.01 for a 10-day interval; IRR 1.001, 95% CI 1.000-1.002; *P*=.04 for a 15-day interval), self-harm (IRR 1.009, 95% CI 1.001-1.016; *P*=.02 for a 5-day interval; IRR 1.005, 95% CI 1.000-1.009; *P*=.04 for a 10-day interval; IRR 1.004, 95% CI 1.000-1.008; *P*=.004 for a 15-day interval), and suicide method (IRR 1.017, 95% CI 1.000-1.034; *P*=.04 for a 1-day interval; IRR 1.007, 95% CI 1.001-1.013; *P*=.003 for a 5-day interval) were identified as being significant.

**Table 2:** Results from Poisson regression analyses of the associations between relative search volumes of suicide-related search terms and the number of suicides for each time interval (1 day, 5 days, 10 days, and 15 days).

Population, suicide-related search terms statistically significant for least one time interval			Sum of 1-day-interval number of suicides, IRR^a^ (95% CI)		Sum of 5-day-interval number of suicides, IRR (95% CI)		Sum of 10-day-interval number of suicides, IRR (95% CI)		Sum of 15-day-interval number of suicides, IRR (95% CI)
**Males**									
	Dropout	1.021 (1.010 to 1.033)^b^		1.004 (1.000 to 1.007)^b^		1.001 (0.999 to 1.003)		1.001 (1.000 to 1.003)	
	Mental health center	1.007 (0.998 to 1.016)		1.004 (1.000 to 1.007)		1.003 (1.000 to 1.006)^b^		1.003 (1.000 to 1.005)^b^	
	Self-harm method	1.010 (1.001 to 1.019)^b^		1.002 (0.998 to 1.006)		1.001 (0.997 to 1.002)		1.000 (0.998 to 1.002)	
	Acetaminophen	1.009 (0.999 to 1.018)		1.002 (0.999 to 1.006)		1.002 (1.000 to 1.004)^b^		1.002 (1.000 to 1.003)	
**Females**									
	Self-harm	1.038 (1.009 to 1.068)^b^		1.013 (1.003 to 1.022)^b^		1.008 (1.002 to 1.014)^b^		1.006 (1.000 to 1.011)^b^	
	Academic score	0.989 (0.978 to 1.000)		0.994 (0.990 to 0.998)^b^		0.997 (0.995 to 1.000)^b^		0.997 (0.995 to 0.999)^b^	
	Dropout	1.015 (1.004 to 1.025)^b^		1.003 (1.000 to 1.005)		1.002 (0.999 to 1.003)		1.001 (1.000 to 1.002)^b^	
	Suicide prevention	1.015 (1.003 to 1.026)^b^		1.003 (0.999 to 1.007)		1.002 (0.999 to 1.009)		1.001 (1.000 to 1.003)	
	Acetaminophen	1.017 (1.000 to 1.019)^b^		1.002 (0.999 to 1.005)		1.000 (0.999 to 1.001)		1.001 (0.999 to 1.002)	
	Domestic violence	0.984 (0.969 to 0.999)^b^		0.999 (0.994 to 1.003)		1.000 (0.997 to 1.002)		0.999 (0.997 to 1.001)	
	Suicide prevention center	1.000 (–0.978 to 1.020)		1.009 (1.001 to 1.016)^b^		1.004 (0.999 to 1.009)		1.003 (0.999 to 1.007)	
**Total population**									
	Dropout	1.017 (1.008 to 1.025)^b^		1.004 (1.001 to 1.006)^b^		1.002 (1.000 to 1.003)^b^		1.001 (1.000 to 1.002)^b^	
	Self-harm	1.017 (0.994 to 1.040)		1.009 (1.001 to 1.016)^b^		1.005 (1.000 to 1.009)^b^		1.004 (1.000 to 1.008)^b^	
	Suicide method	1.017 (1.000 to 1.034)^b^		1.007 (1.001 to 1.013)^b^		1.003 (0.999 to 1.007)		1.003 (1.000 to 1.006)	
	Suicide prevention	1.011 (1.004 to 1.019)^b^		1.002 (0.999 to 1.004)		1.001 (1.000 to 1.003)		1.001 (0.999 to 1.001)	
	Acetaminophen	1.011 (1.001 to 1.015)^b^		1.001 (0.999 to 1.003)		1.001 (0.999 to 1.002)		1.000 (0.999 to 1.001)	
	Call for life	0.995 (0.988 to 1.002)		0.997 (0.994 to 0.999)^b^		0.996 (0.996 to 1.000)		0.999 (0.998 to 1.001)	
	Hanging	1.006 (0.995 to 1.015)		1.001 (0.996 to 1.005)		1.004 (1.001 to 1.006)^b^		1.001 (0.998 to 1.003)	

^a^IRR: incidence rate ratio.

^b^Statistically significant values at *P*<.05.

### Cross-Correlation Analysis

Table 3 lists the cross-correlation coefficients for the relationship between the search volumes of the selected search terms in the previous step and the daily number of suicides for each time lag. In male adolescents, the term dropout presented the highest correlation coefficient for a time lag of 0 days (*r*=0.113). There was no statistically significant correlation for the term mental health center between the time lags of +10 days and +15 days, which were time intervals identified in the Poisson regression analyses. In female adolescents, the term dropout also presented the highest correlation coefficient for a time lag of 0 days (*r*=0.109). The term self-harm showed the strongest correlations for time lags of 0 days and +1 day (both *r*=0.135). The term academic score presented negative associations with the number of suicides in females in the prior analysis, showing the strongest correlation for a time lag of –12 days (*r*=–0.057). For the total population, the terms dropout and suicidal method showed the strongest correlations for a time lag of 0 days (*r*=0.109 and *r*=0.106, respectively). The term self-harm presented the strongest correlation for a time lag of +7 days (*r*=0.103) between time lags of +5 days and +15 days.

**Table 3:** Cross-correlation coefficients for daily number of suicides and relative search volumes of suicide-related search terms.

Time lag (days)	Males (*r* value)		Females (*r* value)			Total population (*r* value)		
	Dropout	Mental health center	Dropout	Self-harm	Academic score	Dropout	Self-harm	Suicidal method
–15	0.022	0.011	0.044	0.072^a^	–0.017	0.044	0.092^a^	0.034
–14	0.036	–0.009	0.038	0.063^a^	–0.009	0.038^a^	0.074^a^	0.002
–13	0.033	0.029	0.022	0.053^a^	–0.032	0.022	0.061^a^	0.011
–12	–0.012	0.013	0.030	0.053^a^	–0.057^b^	0.030	0.052^a^	0.033
–11	–0.020	0.011	0.042	0.073^a^	–0.023	0.042	0.064^a^	0.043
–10	–0.004	0.020	0.056^a^	0.077^a^	–0.022	0.056	0.071^a^	0.052^a^
–9	0.013	0.038	0.047^a^	0.075^a^	–0.023	0.047	0.076^a^	0.055^a^
–8	0.032	0.015	0.077^a^	0.010^a^	–0.001	0.077^a^	0.098^a^	0.053^a^
–7	0.056^a^	0.024	0.088^a^	0.090^a^	0.008	0.088^a^	0.075^a^	0.012
–6	0.026	0.043	0.066^a^	0.049^a^	–0.044	0.066^a^	0.052^a^	0.019
–5	0.033	0.014	0.054^a^	0.069^a^	–0.010	0.054^a^	0.057^a^	0.035
–4	0.006	0.032	0.070^a^	0.078^a^	–0.020	0.070^a^	0.062^a^	0.049^a^
–3	0.026	0.013	0.070^a^	0.074^a^	0.008	0.070^a^	0.066^a^	0.061^a^
–2	0.051^a^	0.056^a^	0.064^a^	0.098^a^	–0.034	0.064^a^	0.094^a^	0.091^a^
–1	0.068^a^	0.015	0.090^a^	0.130^a^	0.015	0.090^a^	0.123^a^	0.100^a^
0	0.113^b^	0.053^a^	0.109^b^	0.135^b^	–0.001	0.109^b^	0.106^a^	0.106^b^
+1	0.095^a^	0.012	0.078^a^	0.135^b^	0.019	0.078^a^	0.118^a^	0.097^a^
+2	0.066^a^	0.021	0.066^a^	0.129^a^	–0.016	0.066^a^	0.104^a^	0.065^a^
+3	0.044	0.036	0.067^a^	0.112^a^	–0.003	0.067^a^	0.085^a^	0.056^a^
+4	0.050^a^	0.033	0.060^a^	0.107^a^	–0.019	0.060^a^	0.090^a^	0.046
+5	0.063^a^	0.027	0.044	0.102^a^	–0.027	0.044^a^	0.088^a^	0.055^a^
+6	0.053^a^	0.027	0.074^a^	0.118^a^	0.002	0.074^a^	0.103^b^	0.051^a^
+7	0.067^a^	0.072^a^	0.089^a^	0.111^a^	0.013	0.089^a^	0.076^a^	0.029
+8	0.042	0.019	0.068^a^	0.083^a^	0.014	0.068^a^	0.067^a^	0.020
+9	0.029	0.025	0.047^a^	0.095^a^	–0.007	0.047^a^	0.087^a^	–0.005
+10	0.020	0.020	0.056^a^	0.101^a^	0.012	0.056^a^	0.081^a^	–0.004
+11	–0.007	0.033	0.044	0.103^a^	–0.009	0.044	0.080^a^	0.015
+12	0.006	0.013	0.019	0.090^a^	–0.041	0.019	0.069^a^	0.045
+13	0.036	0.024	0.032	0.092^a^	–0.010	0.032^a^	0.084^a^	0.013
+14	0.050^a^	0.018	0.039	0.082^a^	–0.003	0.039^a^	0.065^a^	–0.009
+15	0.022	0.010	0.042	0.075^a^	0.010	0.042^a^	0.058^a^	–0.041

^a^Statistically significant values at *P*<.05.

^b^Highest correlation coefficients for 2 significant time intervals.

## Discussion

### Principal Findings

This study investigates the association between the number of suicide deaths and the internet search volumes of terms related to suicide or self-harm among South Korean adolescents. We found that the internet search volume for dropout was correlated with the number of suicides among South Korean males, females, and the total population. In males, dropout was the only internet search term that showed a significant correlation with the number of suicide deaths. In females, the search volumes for self-harm were positively correlated while those for academic score were negatively correlated with the number of suicides. In the total population, the search volumes for self-harm and suicidal method were correlated with the number of suicides. However, the correlation between the search volumes and the number of suicides was relatively weak across all population groups (IRR 0.990-1.068). The estimated time lags of the increasing trends between suicide death and search volumes were shorter than expected, with a time lag of 0 days predominating. To our knowledge, our study is the first to investigate the association of the actual daily number of suicides with the internet search volumes of terms related to suicide or self-harm.

In the analyses of the correlations between the internet search terms, we found differences between male and female adolescents. In terms of the overall correlation between the search terms, the correlations between the terms were stronger for female adolescents than male adolescents. Previous studies have shown that the purpose of internet use differs between genders, with this also differing between countries [33,34]. Our correlation results might be due to female adolescents using the internet more for information searching or social networking services compared with males who mainly use the internet for web-based gaming, as reported in previous studies [33,34]. In male adolescents, a significant within-group correlation was found for suicide-related terms and self-harm–related terms. In females, there was also a significant within-group correlation for suicide-related terms and self-harm–related terms, while a significant intergroup correlation was also observed between suicide-related terms and self-harm–related terms. This finding is consistent with previous findings [35-37] of self-harm and suicide attempts being much more closely associated with female than male adolescents. Previous suicide attempts and self-harm are known to be the most important risk factors for suicide death [38,39]. Therefore, the stronger correlation between suicide-related terms and self-harm–related terms in female adolescents might indirectly indicate that they have a higher risk of suicide death compared with male adolescents. In addition, the search trends of suicide prevention terms were correlated with those of suicide risk factor terms and suicide-related terms in females. Considering that young females avail psychiatric services more often than males do [40], our finding suggests that among adolescents who died by suicide, females were more likely to have sought help. The suicide prevention effect was found to be stronger in female than in male adolescents [41]. Thus, providing active treatment to female adolescents seeking help may be effective for suicide prevention, whereas screening to detect suicide risk may be beneficial for male adolescents (unlike in females).

In all 3 population groups, the internet search term dropout was correlated with the number of suicides. Dropping out of school is strongly associated with suicidality [42]. Further, truancy is associated with suicide and self-harm [42] and failing coursework [43] is associated with suicidal ideation. Our finding is consistent with previous reports on the association between school absenteeism and suicidality among students. Although the data in our study were collected using teacher reports, and hence, the adolescents who died by suicide were in school and had not actually dropped out, they could indirectly represent the students who were thinking of dropping out. Therefore, suicide risk assessments of students considering dropping out of school may be helpful for suicide prevention among students.

In males, the internet search term mental health center was positively correlated with the number of suicides at time intervals of 10 days and 15 days. However, there was no time lag showing a significant correlation in the cross-correlation analysis; this might be because the RSV for the search term mental health center was mostly 0 (1290 of 1827 days), which might have interfered with the analysis results. In females, the search terms self-harm and academic score were correlated with the number of suicides. Self-harm is widely known to be a significant risk factor for suicide in adolescents [44-46]. In addition, considering that self-harm is more common in female adolescents [47], this association might have been more pronounced in females than that in males. In particular, the search term academic score was the only search term with a negative correlation in all population groups. Previous studies [43,48,49] have shown that low school grades and academic performance were associated with suicide in youths. South Korea is well-known for its stressful competitive education environment, which adversely affects the emotional and physical well-being of adolescents [50]. For these reasons, academic stress in South Korean adolescents shows strong correlations with suicidal ideation and suicide attempts [51,52]. However, the search term academic score showed the strongest negative correlation with the daily number of suicides for a time lag of –12 days. This might mean that adolescents searching the internet for information related to their academic concerns might indirectly reduce their suicidal behaviors. In other words, it can be considered that interest in academic information could be a protective factor for suicide. Further, this study does not include adolescents who were not attending school, who are known to have higher suicide rates than active students [53], which may have influenced the negative correlation between the search volume for academic score and the number of suicides.

In the total population, the association between the number of suicides and the search volume was similar to that observed in females. This similarity was also demonstrated in the analysis of the correlation between search terms, which might indicate that females perform internet searching mainly. The search term suicide method was correlated with the number of suicides only in the total population. As reported previously, internet use related to suicide or self-harm is thought to increase the likelihood of suicide and self-harm [54,55], and the risk of suicide is higher for methods of self-harm that are more violent [56].

One difference between our study and previous studies was that the internet search term depression was not associated with the number of suicides in any population group. Various studies conducted in the United States [8], United Kingdom [12,57,58], Ireland [14], Japan [9], and Taiwan [10] have found positive correlations between search activity related to the term depression and suicide rates/numbers. In contrast, a multinational study [13] found no correlation between the search volume for depression and suicides or not even a negative correlation in some countries. Previous studies have shown that the findings might differ depending on population sizes and the national suicide prevention policies. In South Korea, various suicide prevention policies have been implemented since 2011, which include internet monitoring [59]. According to this policy, when anyone searches for terms related to suicide or depression on the internet, the home page of the Korea Suicide Prevention Center (which is supported directly by the government) will be displayed. This preventive policy might reduce the suicide risk of adolescents by preventing access to web pages related to suicide or depression [60]. However, the search volume for depression increased significantly 1 day after the suicide death of a famous Korean singer, which may have influenced the association with the number of suicide deaths [16].

Our analysis of the time lags between the number of suicides and the search volume revealed that this was most commonly 0 days, demonstrating a close correlation between internet searching trends and the actual suicide activity. This might reflect the impulsiveness of adolescent suicide, which differs from the characteristics of suicide in other populations. Previous studies showed that the correlation between news reports about suicide death of celebrities and the number of suicide deaths was the strongest within time intervals from 3 days to 10 days [9,16,17], which contrasts with the generally shorter time interval among adolescents in our study. However, when considering the relatively weak correlation for the entire search term, our results need to be interpreted with caution. Previous studies using Google Trends [13,61] have also mentioned the negative outcomes and limitations of using search trends for behavioral forecasting. These studies have indicated that specific search activities may not be directly linked to the actual behavior; different population groups do not perform search activities in the same way, and specific situations could decrease the accuracy of search trends obtained by RSV, thereby lowering their reliability.

Nevertheless, unlike previous studies, our study has some meaningful implications in that it focused solely on children and adolescents as the participants. By controlling the target population to children and adolescents, we estimated the direct internet search activity specifically for adolescents rather than their indirect exposure via media. This might be because adolescents have better access to the internet than other population groups, which leads to more active search activity. Therefore, a surveillance system that responds immediately to search activities related to suicide and self-harm in adolescents may be needed.

### Limitations

This study has several limitations. First, the significant correlation between internet search volumes and the number of adolescent suicides does not infer the presence of an antecedent relationship between the searching of terms related to suicide/self-harm and suicide. In the same context, the association in our research results was relatively weak, with an IRR close to 1. This might be a characteristic of observational epidemiological studies, but it is also a limitation of our research. Second, our study includes adolescent suicides reported by schools and does not include out-of-school adolescents; hence, our findings cannot represent all the suicides among South Korean adolescents. However, in South Korea, education is compulsory up to the middle-school level, and the dropout rate of high-school students in 2021 was only 1.5% [25], which means that the sample in this study represents almost all adolescents in South Korea. Third, we did not compare the association between the internet search volume and the number of suicides by categorizing the search methods as personal computers and mobile devices, and hence, this should be investigated accordingly in future research. Fourth, we did not adjust for external factors such as weather, economic conditions, or celebrity suicides that could influence suicide rates in children and adolescents. Fifth, we could not include searches performed by adolescents who did not log into the internet search engine. These limitations may have affected the identified correlations between the search volumes and the number of suicides, perhaps leading to false-positive results. Sixth, we did not include universal terms related to suicide/self-harm used globally, but we only used search terms that consider the characteristics of suicide and self-harm in South Korean adolescents [24,28]. For example, search volumes for other methods of suicide and self-harm, such as firearm use, were not included in the analysis. Therefore, there are limitations to generalizing our research findings globally.

### Conclusions

In this study, we identified associations between the internet search volumes of terms related to suicide/self-harm and the number of South Korean adolescent suicides, with gender differences in these associations. The time lag with the strongest correlation between the internet search volume and suicide death was very short. Given that adolescent suicide has multidimensional risk factors, the search volume does not directly indicate the cause of suicide, but it can indirectly predict the increasing trend in the number of suicides as the search volume increases. However, the correlation between the number of suicide deaths and internet searches was relatively weak, and there are still limitations in utilizing our findings in real world. Further research will be necessary in the future to establish a sensitive internet surveillance system.

## Appendix

Multimedia Appendix 1 Standard Korean version of the search terms related to suicide and self-harm.

## Abbreviations

IRR: incidence rate ratio
RSV: relative search volume

## Data Availability

This study includes data collected from the Korean Ministry of Education and requires permission from the Korean Ministry of Education to access it. Requests to access these data sets should be directed to HJH, the corresponding author.
