# Supplementary material for: Association Between Internet Searches Related to Suicide/Self-harm and Adolescent Suicide Death in South Korea in 2016-2020: Secondary Data Analysis
Source: J Med Internet Res. 2023 Apr 20;25:e46254. doi: 10.2196/46254 (PMC10160929; doi:10.2196/46254)
Supplement: Multimedia Appendix 1 [file jmir_v25i1e46254_app1.docx]

**Table S1. Standard Korean version of the search terms related to suicide and self-harm**

| Classification of search terms | Search terms | Korean version of search terms | Synonyms | Korean version of Synonyms |
| --- | --- | --- | --- | --- |
| Suicide-related terms | Suicide | 자살 | Suicide | 자살 |
|  | Suicide method | 자살방법 | Suicide method  How to suicide  How to commit suicide | 자살방법  자살하는법  자살하는방법 |
|  | Dying method | 죽는방법 | Dying method  How to die | 죽는방법  죽는법 |
|  | Suicidal idea | 자살충동 | Suicidal idea  Suicidal thinking | 자살충동  자살생각 |
|  | Fall-down | 투신 | Fall down  Suicide by jumping from a height | 투신  투신자살 |
|  | Hanging | 목맴 | Hanging  Hanging suicide  Neck hanging | 목맴  목맴자살  목매달기 |
|  | Will | 유서 | Will  Hot to write will | 유서  유서쓰는법 |
| Self-harm-related terms | Self-harm | 자해 | Self-harm | 자해 |
|  | Self-harm method | 자해방법 | Self-harm method  How to self-harm | 자해방법  자해하는법 |
|  | Wrist cutting | 손목자해 | Wrist cutting  How to cut my wrist  Wrist cutting method | 손목자해  손목자해하는법  손목자해방법 |
|  | Self-harm wound | 자해흉터 | Self-harm wound  Self-harm mark  Treatment for Self-harm wound | 자해흉터  자해자국  자해흉터치료 |
|  | Drug overdose | 약물과다복용 | Drug overdose  Drug lethal dose | 약물과다복용  약물치사량 |
|  | Acetaminophen | 타이레놀 | Acetaminophen overdose  Acetaminophen lethal dose | 타이레놀과다복용  타이레놀치사량 |
| Suicide risk factor terms | Academic score | 성적 | Academic score  Academic concern | 성적  성적고민 |
|  | Bullying | 왕따 | Bullying  Covert bullying  Outcast | 왕따  은따  따돌림 |
|  | School violence | 학교폭력 | School violence  School vio | 학교폭력  학폭 |
|  | Family troubles | 가정불화 | Family troubles | 가정불화 |
|  | Domestic violence | 가정폭력 | Domestic violence | 가정폭력 |
|  | Dropout | 자퇴 | Dropout  How to dropout  Dropout method | 자퇴  자퇴하는법 |
|  | Career | 진로 | Career  Career concern | 진로  진로고민 |
| Suicide prevention terms | Suicide prevention | 자살예방 | Suicide prevention | 자살예방 |
|  | Call for life | 생명의전화 | Call for life  Call for life of Korea | 생명의전화  한국생명의전화 |
|  | Suicide prevention center | 자살예방센터 | Suicide prevention center  1393 | 자살예방센터”  1393 |
|  | Psychiatry | 정신과 | Psychiatry  Neuropsychiatry  Psychiatry department  Mental hospital | 정신과  신경정신과  정신건강의학과  정신병원 |
|  | Mental health center | 정신건강복지센터 | Mental health center | 정신건강복지센터 |
| Depression-related terms | Depression | 우울증 | Depression  Depressed  Depressive disorder  Depressive symptom | 우울증  우울  우울장애  우울증상 |
